# Temperature-Dependent Effects on Cyclic Fatigue Resistance in Three Reciprocating Endodontic Systems: An In Vitro Study

**DOI:** 10.3390/ma18050952

**Published:** 2025-02-21

**Authors:** Marcela Salamanca Ramos, José Aranguren, Giulia Malvicini, Cesar De Gregorio, Carmen Bonilla, Alejandro R. Perez

**Affiliations:** 1 Department of Endodontics, Rey Juan Carlos University, 28032 Madrid, Spain; marce0415@gmail.com (M.S.R.); josearanguren@hotmail.com (J.A.); cesargre@me.com (C.D.G.); 2 Unit of Endodontics and Restorative Dentistry, Department of Medical Biotechnologies, University of Siena, 53100 Siena, Italy; giulia.malvicini@student.unisi.it; 3 Nova Southeastern University College of Dental Medicine, Davie, FL 33314, USA; mbonilla1@nova.edu; 4 Surpreendente Research Group, Vila Nova de Gaia 4400-239, Portugal

**Keywords:** cyclic fatigue resistance, nickel–titanium, reciprocating files, temperature effect, thermal treatment

## Abstract

This study aimed to analyze the effect of 1% sodium hypochlorite (NaOCl) solution at different temperatures on endodontic file resistance to cyclic fatigue. A total of 90 files, Reciproc NiTi M-Wire^®^ (REC) (n = 30), WaveOne Gold^®^ (WOG) (n = 30), and Reciproc Blue^®^ (RB) (n = 30) were activated under constant irrigation with 1% NaOCl at 5, 37, and 60 °C in a stainless-steel artificial canal (curvature angle = 60°; radius = 5 mm). The time to the fracture and the maximum and minimum load were recorded for each instrument, and data were subjected to statistical analysis. A higher number of cycles to fracture at 5 °C was observed between WOG and RB compared to the REC system (*p* < 0.05). The RB files were more resistant to cyclic fatigue fracture at 60 °C than WOG and REC (*p* < 0.05). No statistically significant differences were found between the mean values of the three file types used at 37 °C. The high temperature of NaOCl significantly affects the lifespan of endodontic files, making them more prone to fractures due to cyclic fatigue. The files manufactured with heat treatment showed a longer life than M-wire reciproc files.

## 1. Introduction

Reciprocating motion is a movement strategy used in endodontic instrumentation that involves alternating clockwise and counterclockwise rotation. Compared to continuous rotation, reciprocating kinematics has shown several benefits, including increased resistance to cyclic fatigue, reduced torsional stress, and reduced working times [1,2,3]. This is because reciprocation generates less stress on the instrument than continuous rotation, reducing the risk of fatigue failure. Furthermore, the alternating motion prevents excessive stress on the instruments during apical binding, as the unwinding action ensures that the angles remain below the critical values for torsional fractures [4,5,6]. This technique has consistently performed well across varying skill levels, demonstrating its versatility and dependability, even when used by less experienced clinicians [7,8].

Many reciprocating files made from different shapes, materials, and alloys have been marketed and tested in numerous studies [4,6,7,9,10,11,12]. Reciproc (VDW, Munich, Germany) was the first commercially available system developed with an M-wire NiTi alloy designed for reciprocating motion. Later, the development of Reciproc Blue (VDW), made from thermally treated alloys, significantly improved flexibility and resistance to fracture in these systems [4,9].

The blue heat treatment reduces the shape memory of the NiTi alloy and induces the occurrence of martensitic transformation into two phases, increasing fatigue strength and flexibility compared to conventional M-wire [13].

Despite these improved mechanical properties, reciprocating files can still fracture due to cyclic fatigue, especially when navigating through root canals with unfavorable curvature angles for the NiTi alloy. Cyclic fatigue occurs when the file undergoes alternating tension and compression cycles (cycle–tension–compression) [14]. These cycles are responsible for fatigue and the potential for fracture [15].

During these alternating cycles, the alloy of the NiTi file undergoes mechanical transitions from a martensitic to an austenitic phase, which occurs because of a stress application and/or temperature decrease. Indeed, the reversible part of this transition involves a structure-dependent temperature increase specific to each alloy [16,17]. Conventional NiTi alloys remain in the austenitic phase (characterized by hardness and strength) at intracanal temperature (35 °C), exhibiting high hardness, rigidity, and greater subsceptibility to cyclic fatigue and fracture [16]. In contrast, at lower temperatures, it mainly consists of martensite, which is softer and more flexible [15,18].

An alternative clinical approach to mitigate cyclic fatigue and fracture risk is to lower the temperature in the root canal by using a pre-cooled irrigation solution [19,20]. This promotes the transition to the more flexible martensitic stage. Another alternative is to induce structural changes in the alloy through temperature fluctuations that adapt to the temperature in the root canal. This approach ensures adequate flexibility and pseudoelasticity during rotary instrumentation and increases resistance to cyclic fatigue [20]. Indeed, new rotary and reciprocating endodontic files undergo treatment during their manufacturing process to make them more flexible according to these principles [15,20,21,22,23,24,25,26,27,28].

However, in contrast to the use of pre-cooled solutions, it is common practice among clinicians to use pre-heated irrigants [21,28,29] to improve antibacterial properties and promote tissue dissolution during root canal treatment. While this practice may improve the antibacterial effect, it overlooks parameters that could prevent cyclic fatigue and file fracture [21].

Numerous studies have compared the fatigue resistance of different rotary and reciprocating endodontic instruments at ambient temperature [11,30,31,32,33,34,35]. However, few studies consider the actual working temperature of these files, which can significantly affect their resistance to cyclic fatigue and overall performance [17,19,21,32,36,37]. De Vasconcelos et al. evaluated the effect of two different temperatures (i.e., 20 °C and body temperature), demonstrating that an increase in temperature leads to a decrease in the NCF. Similarly, Shen et al. reported that the fatigue resistance of NiTi files decreases when the temperature increases from 0 °C to 60 °C [20]. Nevertheless, the experiment was conducted in water, which does not reproduce clinical conditions. Recently, a study from Alfawaz et al. tested the effect of heating a NaOCl solution up to 60 °C, demonstrating accelerated instrument fractures [17]. However, this study focused on ProTaper Gold instruments (Dentsply Sirona, York, PA, USA) with continuous rotation, while only limited evidence exists on reciprocating motion [38]. Despite these data, the literature lacks evidence comparing the effect of different temperature ranges on the cyclic fatigue resistance of reciprocating instruments. Therefore, no effective protocol exists to balance the mechanical performance of reciprocating files with the optimal NaOCl temperature.

This study aimed to evaluate the changes that occur using three types of reciprocating files at three different temperatures (low, physiological, and high), including time to fracture, number of cycles to fracture, and maximum load. This evaluation is performed using an established and documented model.

## 2. Materials and Methods

### 2.1. Endodontic Reciprocating Instruments

This study evaluated three endodontic reciprocating systems: WaveOne Gold^®^ (Dentsply, Maillefer, Ballaigues, Switzerland), Reciproc, and the Reciproc Blue^®^ (VDW, Munich, Germany) files. All tested files had a standardized length of 25 mm and a tip diameter of 0.25 mm, differing primarily in their cross-sectional shape and taper. WaveOne Gold^®^ had a parallelogram-shaped cross-section with a variable taper of 0.07 mm/mm over the first 3 mm of the tip. In contrast, the Reciproc and the Reciproc Blue^®^ files had an “S”-shaped cross-section with a variable taper of 0.08 mm/mm over the same 3 mm tip. Following the manufacturer’s instruction, the WaveOne Gold^®^ operated a reciprocating movement (150°–30°) with an angular speed of 350 rpm. Meanwhile, both types of Reciproc^®^ files (Reciproc^®^ and Reciproc Blue^®^) functioned with a reciprocating movement (150°–30°) at 300 rpm.

### 2.2. Study Design and Samples

Ninety files (thirty per system) were used in this study and randomly assigned to three temperature groups: 60 °C (Group A), 37 °C (Group B), and 5 °C (Group C) with 10 files in each group. These conditions were designed to simulate real clinical situations where the irrigant is pre-cooled, heated, or at intra-canal temperature. Each file was tested once to the point of fracture, following the specifications explained below. The recorded variables included the maximal load (ML), time to fracture (TTF), and NCF. The NCF was calculated using the recorded TTF and the angular speed specified by the manufacturer’s instructions.

### 2.3. Cyclic Fatigue Testing Device

Cyclic fatigue was assessed using a customized device, described by Larsen et al. [29]. The analyses were conducted with a universal force transducer INSTRON 3345 (Instron, Norwood, MA, USA), which is capable of measuring force, displacement, and strain within a range of 0.1 N to 5 kN and an accuracy of ± 0.5%. This device allows for cyclic loading tests, tensile, and compressive testing, and its main limitations include a limited maximum load capacity of 5 kN and a maximum crosshead speed of 1000 mm/min. The results were visualized using the Bluehill Lite interface software (v2.18, 2005).

### 2.4. Artificial Canals and Irrigant Preparation

**Figure 1:**
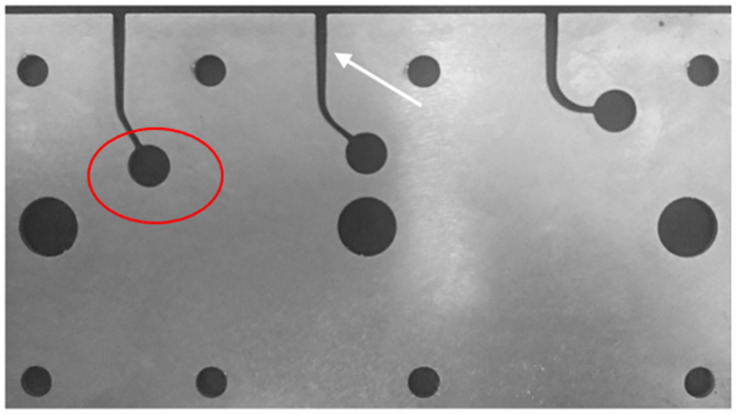
A stainless-steel block was designed and manufactured to simulate root canals and perform cyclic fatigue analyses of three types of endodontic files at three different temperatures. The artificial canals are indicated with a white arrow (26 mm × 1.5 mm; angle = 60°; curvature radius = 5 mm), and the reservoir holes for the irrigant solution are marked with a red circle.

The artificial root canals were manufactured from a stainless-steel block (Figure 1) to create canals measuring 26 mm × 1.5 mm with a curvature angle of 60° and a radius of curvature of 5 mm. A reservoir of 1% NaOCl irrigating solution was placed at the end of each artificial canal (Figure 1). The block was cleaned, and the canals were lubricated with a spray universal lubricant (KaVo Dental Technologies, Charlotte, NC, USA) to minimize instrument friction. The block was then covered with transparent acrylic material to facilitate observation during the experiment. In addition, the entire stainless-steel block and files were immersed in a cuvette containing 1% NaOCl at the desired temperature for at least 1 min before testing. The reservoir holes in the acrylic cover were sealed with a cannulated rubber plug to retain the irrigant solution from a 20-cc syringe at 5, 37, or 60 °C.

### 2.5. Instrumentation Procedure

During the procedure, the files were positioned 0.5 mm from the entrance of the artificial canal to prevent contact with the device once the working length (22 mm) was reached. Instrumentation was executed using a (6:1) reduction handpiece powered by an endodontic motor (WaveOne^®^, Dentsply-Maillefer, Ballaigues, Switzerland) and mounted on the force transducer’s universal stainless-steel support. The instruments aligned with the artificial canal entrance, and the system activated.

Once activated, the files advanced into the artificial canal at a constant speed of 23.5 mm/sec, reaching the established working length in approximately 1 min. Under these conditions, the instrument could rotate freely within the canal. Each essay continued until fracture. At this point, the corresponding data on time, forces, and maximum load were recorded for each file. The same operator conducted all the experimental procedures to minimize bias.

**Figure 2:**
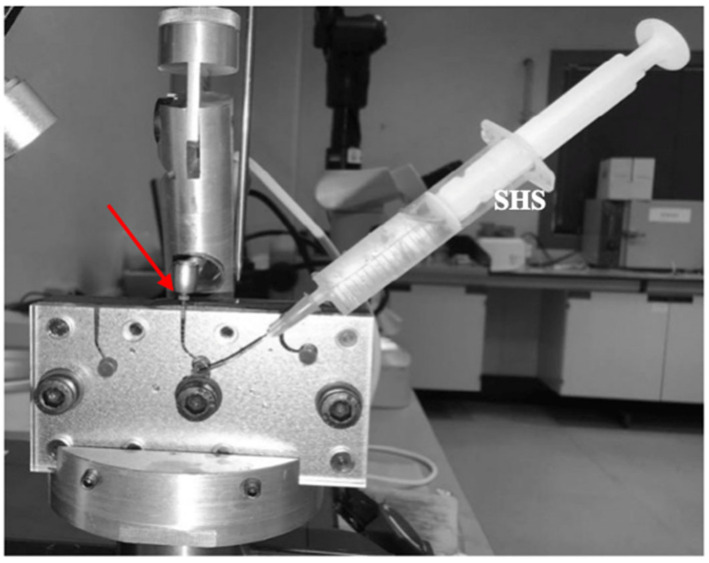
Experimental assembly of the device for cyclic fatigue analysis of endodontic files. The figure highlights the file’s disposition inside a simulating root canal (red arrow), the sodium hypochlorite-containing syringe (SHS), and the reduction handpiece powered by a micromotor.

Figure 2 shows the experimental assembly of the customized device used for cyclic fatigue analysis. 

### 2.6. Statistical Analyses

The results were analyzed using STATISTICA v5.0 (StatSoft Inc., Tulsa, OK, USA). An unpaired Student’s t-test for small samples determined the statistical significance of the mean NCF values for each treatment. All statistical analyses were conducted using a 95% confidence level.

An analysis of variance (ANOVA) was performed for comparisons involving multiple treatments, considering NCF and maximum load as dependent variables. A two-way ANOVA was conducted with file type and irrigant temperature as independent variables. A Levene’s test was used to assess the homogeneity of variance, ensuring the validity of the analysis. A Tukey’s HSD (Honestly Significant Difference) test was applied for variance comparisons, enabling multiple comparisons. The significance level was set at 5%.

## 3. Results

Table 1A,B show the results of statistical analyses examining the effects of file type and irrigation solution temperature on the NCF. The results from the two-way ANOVA revealed that both file types [F (2,81) = 7.45, *p* = 0.001] and irrigant temperature [F (2,81) = 43.25, *p* = 0.000] significantly influence the NCF. Among the three file systems, Reciproc Blue^®^ exhibited the highest mean NCF, followed by WaveOne Gold^®^, while Reciproc ^®^ files had the lowest NCF.

The Tukey’s HSD test revealed no significant difference (*p* > 0.05) between the mean NCF values of Reciproc Blue^®^ and WaveOne Gold^®^. In contrast, a statistically significant difference was observed between Reciproc Blue^®^ and Reciproc^®^ (*p* < 0.05).

**Figure 3:**
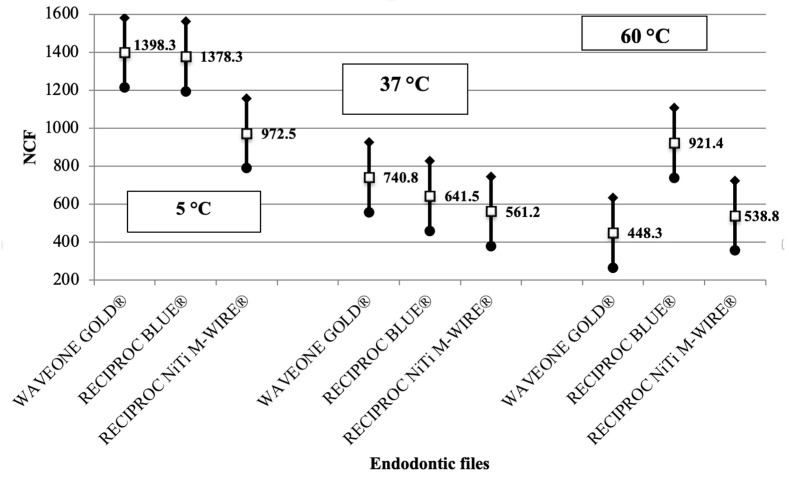
Tukey 95% confidence intervals of NCF. (**□**) Mean of NCF; (●) lower limit and (♦) upper limit of 95%CI.

**Figure 4:**
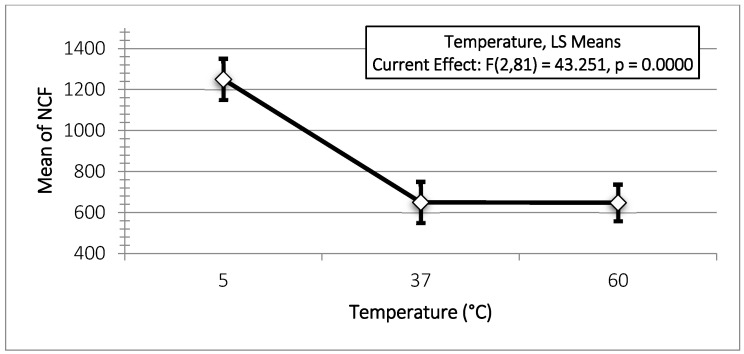
Mean of NCF values according to temperature (°C). Vertical bars denote the 0.95 confidence intervals.

**Figure 5:**
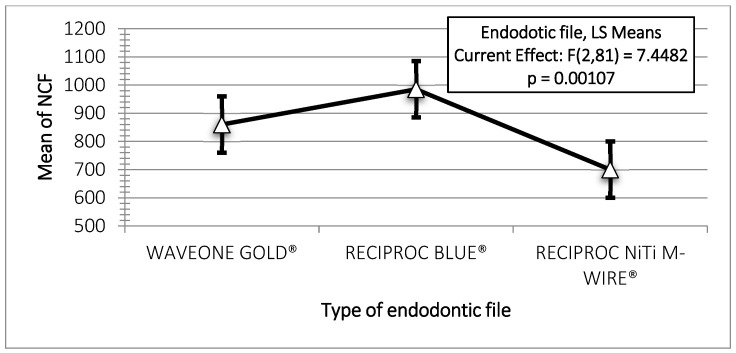
Mean of NCF values according to the type of endodontic files. Vertical bars denote the 0.95 confidence intervals.

Regarding the relationship between NCF and temperature, a significant increase in NCF was observed when mechanical instrumentation of the artificial canals was conducted at 5 °C (*p* < 0.05). However, the difference between 60 ° and 37 °C was not statistically significant (*p* > 0.05) for any instrumentation system (Table 1B, Figure 3 and Figure 4). Reciproc Blue^®^ demonstrated the highest performance under cyclic fatigue at 60 °C among the three endodontic systems (Figure 5). Specifically, its mean NCF significantly exceeded the WaveOne Gold^®^ (*p* < 0.05). Conversely, no statistically significant differences were found between WaveOne Gold^®^ and Reciproc^®^ (*p* > 0.05) or between Reciproc Blue^®^ and Reciproc^®^ systems (*p* > 0.05).

**Figure 6:**
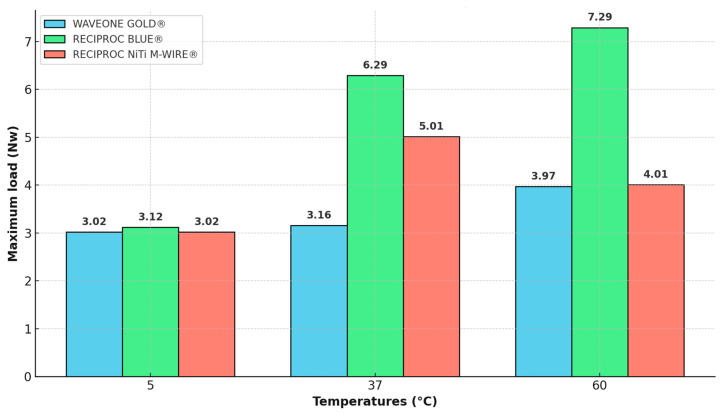
Behavior of maximum load from the different types of files with the temperature of the irrigant solution.

The maximum load of the files at varying temperatures is shown in Figure 6. Notably, distinct behaviors were observed, influenced by file type and temperature. An increasing trend in the maximum load was observed as the irrigant solution temperature raised. The highest maximum load for WaveOne Gold^®^ and Reciproc Blue^®^ was recorded at 60 °C, while Reciproc^®^ reached its peak value at 37 °C. However, no statistically significant differences (*p* > 0.05) were observed in the mean maximum load across different combinations of file types and temperatures during instrumentation.

## 4. Discussion

The present in vitro study investigated the effect of a 1% sodium hypochlorite at controlled temperatures (i.e., 5 °C, 37 °C, and 60 °C) on the cyclic fatigue resistance of three endodontic files systems: Reciproc ^®^, WaveOne Gold^®^, and Reciproc Blue^®^.

This study’s findings highlight the significant impact of temperature on the cyclic fatigue resistance of endodontic files. At lower temperatures (e.g., 5 °C), the files exhibited higher NCF, indicating greater resistance to cyclic fatigue. These results are in accordance with previous studies [19,37] that lower temperatures promote the transition to the more flexible martensitic phase of the NiTi alloy. This transition prevents cyclic fatigue and file fracture during endodontic procedures.

NiTi exhibits a martensitic phase at lower temperatures that is more flexible and less susceptible to fracture. At higher temperatures, the alloy transforms into an austenitic phase that is stiffer and more prone to cyclic fatigue failure [16]. The findings suggest that working at a lower temperature (e.g., 5 °C) increases the NCF, probably due to the files’ enhanced flexibility and reduced brittleness in their martensitic phase.

Additionally, a correlation between the NCF and temperature was observed (Figure 6), with the NCF increasing as the temperature decreased. These results are consistent with those of previously published studies [19,20,37], all of which reported increased resistance to cyclic fatigue at lower temperatures.

The findings indicate that the differences between mean NCF values of Reciproc Blue^®^ and WaveOne Gold^®^ files were not statistically significant, suggesting that both file systems exhibit comparable durability under cyclic fatigue. However, Reciproc Blue^®^ significantly outperformed Reciproc^®^ regarding NCF, indicating a superior resistance to cyclic fatigue compared to the latter.

The superior performance of Reciproc Blue and WaveOne Gold compared to Reciproc, as well as the evident improvement with temperature change, can be attributed to their unique thermomechanical manufacturing process, particularly the blue heat treatment applied to Reciproc Blue^®^ and the distinct properties of WaveOne Gold^®^. The heat treatment applied to Reciproc Blue^®^ and WaveOne Gold^®^ instruments enhances their fatigue strength and flexibility, enabling them to better withstand the mechanical stress encountered during instrumentation.

The ability of these instruments to maintain their structural integrity under various temperature conditions is crucial for their effectiveness in clinical practice. This differentiation underscores the advancements in material technology and design incorporated in these new systems, which reflect improvements targeted at enhancing fatigue resistance.

In accordance with a recent study [38], the best performance under cyclic fatigue at 60 °C was exhibited by Reciproc Blue^®^, which significantly exceeded the mean NCF of WaveOne Gold^®^ files (Table 2, Figure 3, Figure 5 and Figure 6). This suggests that Reciproc Blue^®^ is more resilient under higher temperature conditions, which is likely attributed to its material composition or design features that reduce the impact of thermal stress on fatigue resistance [38].

The present findings differ from those of previous studies [36,38], demonstrating that Reciproc Blue °C shows the highest cyclic fatigue resistance at 37 °C. In contrast, they demonstrated that WaveOne Gold^®^ shows the highest NCF at 37 °C. These differences may be attributed to differences in experimental designs, sample sizes, and variables assessed (time to fracture versus NCF).

Moreover, the maximum load exhibited an inverse relationship with file flexibility. This trend suggests that file flexibility decreases as the maximum load increases. Thus, the maximum load can be considered an indicator of file flexibility, although other factors, such as temperature, can also influence this property.

At higher temperatures (60 °C), the maximum load capacity increases; however, this does not correspond to statistically significant differences in NCF across all conditions. This suggests that while the files can withstand higher loads before fracturing, the number of cycles to fracture does not increase uniformly.

Although this study did not yield statistically significant results, the maximum load behavior is in accordance with findings from Shen et al. [20], which are characterized by an upward trend with increasing irrigating solution temperature. This trend implies reduced file flexibility at higher temperatures for the irrigating solution.

In the present in vitro study, a NaOCl concentration of 1% was selected. Previous studies have demonstrated that there are no significant differences in disinfection efficacy or treatment success among various concentrations of NaOCl [39,40,41]. The present study aimed to evaluate exclusively the effect of temperature on the instruments rather than the effect of its concentration. Therefore, since the choice of concentration does not substantially affect the outcomes, a 1% NaOCl concentration represents a suitable option to assess the effect of temperature on cyclic fatigue resistance.

From a clinical perspective, recommendations to minimize fracture risks associated with NiTi alloy cyclic fatigue should consider three scenarios. First of all, when enhancing the bactericidal effect of the NaOCl, an irrigant solution is necessary; for example, in necrotic teeth, it is strongly recommended to use Reciproc Blue^®^ files with a NaOCl irrigant solution at 60 °C (NCF = 921.4). This choice ensures an adequate disinfection of the root canal system while minimizing the risk of file fracture.

When using 37 °C NaOCl solution during root canal instrumentation, although no statistically significant differences in NCF values were observed among different files at this temperature, using Wave One Gold^®^ files at 37 °C (NCF = 740.8) seems to reduce the risk of file fracture compared to Reciproc Blue^®^ files (NCF = 641.5) and Reciproc NiTi M-Wire^®^ files (NCF = 561.2), as shown in Figure 3. Thus, Wave One Gold^®^ files can be a safer choice for routine procedures, balancing mechanical performance and disinfection efficiency.

Additionally, for cases requiring prolonged instrumentation or involving complex canal anatomies, the most effective approach is using an irrigant at 5 °C with Wave One Gold ^®^ files (NCF = 1398.3) and Reciproc Blue^®^ (NCF = 1378.3). This strategy could be useful to further minimize the risk of file fracture due to cyclic fatigue during demanding procedures.

This study presents some limitations: First, controlled laboratory conditions using artificial canals do not fully replicate the challenges of natural root canals. Factors such as canal anatomy, dentin hardness, and biological tissue presence may influence endodontic file performance in clinical practice. Second, while the study evaluated the effect of different irrigant temperatures, other variables, such as their concentrations, were not explored. Third, the study used fixed temperatures; however, in real clinical situations, the actual temperature of the irrigant may fluctuate.

The mechanical behavior of the files at the tested temperatures supports the implementation of clinical protocols that minimize fracture risk while optimizing the bactericidal or anti-inflammatory effects of the NaOCl solution, depending on the selected working temperatures. Future studies should validate the reproducibility of these assays and establish correlations between mechanical results and those obtained in natural tooth root canals. Furthermore, future research should focus on developing a mathematical model to predict the cyclic fatigue resistance of endodontic instruments under varying thermal conditions. These models could forecast cyclic fatigue resistance over an entire temperature spectrum, giving the clinician a powerful tool for making clinical decisions and anticipating instrument behavior.

## 5. Conclusions

In the present study, the results obtained were in line with initial expectations. Indeed, the NaOCl temperature and file type significantly affect the cyclic fatigue resistance. The highest cyclic fatigue resistance was observed at 5 °C when using the WaveOne Gold^®^ and Reciproc Blue^®^ files. Overall, heat-treated endodontic instruments outperform M-wire Reciproc files, with Reciproc Blue^®^ exhibiting the highest cyclic fatigue resistance at 60 °C. Key limitations include the in vitro design of the study, which does not fully replicate clinical conditions, and the use of fixed temperatures, without accounting for real-time fluctuations or varying NaOCl concentrations. From a clinical point of view, the use of Reciproc Blue^®^ at 60 °C could enhance bactericidal effects, while WaveOne Gold^®^ at 37 °C or 5 °C irrigation could minimize fracture risk.

## Figures and Tables

**Table 1:** Media and standard error of the files NCF calculated for (A) the different types of endodontic files and (B) for the mechanical instrumentation of artificial root canals at different temperatures.

	**(NCF)** **(A) Effect of the File Types**				
	**Central Tendency** **and Dispersion Measures**			**Confidence Intervals** **(95%CI)**	
Type of endodontic files	**N**	Mean *	**Standard Error**	**Lower limit (LL)**	**Upper Limit (UL)**
WAVEONE GOLD^®^	30	862.5	53.35	756.30	968.60
RECIPROC BLUE^®^	30	980.4 ^a^	53.35	874.22	1086.50
RECIPROC NiTi M-WIRE^®^	30	690.8 ^a^	53.35	584.70	797.00
	**(NCF)** **(B) Effect of temperature**				
	**Central Tendency** **and Dispersion Measures**			**Confidence Intervals** **(95%CI)**	
Temperature of NaOCl (°C)	**N**	Mean **	**Standard Error**	**Lower limit (LL)**	**Upper Limit (UL)**
5	30	1249.70	53.35	1143.5	1355.80
37	30	647.80	53.35	541.7	754.00
60	30	636.20	53.35	530.0	742.30

* Mean of the NCF calculated for all the endodontic files of each type described at all temperatures. ** Mean of the NCF calculated for all endodontic files tested at the temperature described. ^a^ Statically significant differences (*p* < 0.05).

**Table 2:** Mean and standard error of the NCF for the different types of files and temperatures of the irrigant solution used during cyclic fatigue analysis.

Tukey’s HSD (*Honestly Significant Difference*) Test			Endodontic Files NCF		Endodontic Files NCF (95% Confidence Intervals) (95%CI)	
Type of File	Temperature (°C)	N	Mean	Standard Error	Lower Limit (LL)	Upper Limit (UL)
WAVEONE GOLD^®^	5	10	1398.30	92.41	1214.40	1562.10
	37	10	740.80	92.41	557,00	924.71
	60	10	448.30 ^a^	92.41	264.40	632.20
RECIPROC BLUE^®^	5	10	1378.30	92.41	1194.40	1562.10
	37	10	641.50	92.41	457.6	825.40
	60	10	921.40 ^a^	92.41	737.5	1105.30
RECIPROC NiTi M-WIRE^®^	5	10	972.50	92.41	788.6	1156.40
	37	10	561.20	92.41	377.3	745.10
	60	10	538.80	92.41	354.9	722.60

^a^ Statistically significant differences (*p* < 0.05).

## Data Availability

The original contributions presented in this study are included in the article. Further inquiries can be directed to the corresponding author.
